# Supplemental fermented plant product (‘*Manda Koso*’) reduces succinate and deoxycholate, as well as elevates IgA and mucin levels, in rats fed a high-fat diet

**DOI:** 10.3892/br.2015.506

**Published:** 2015-08-24

**Authors:** YONGSHOU YANG, NOVITA VIVI SITANGGANG, YUKAKO OKAZAKI, HIROYUKI TOMOTAKE, KENTARO ARITA, TAKAYUKI ASHIDA, NORIHISA KATO

**Affiliations:** 1 Graduate School of Biosphere Science, Hiroshima University, Higashi-Hiroshima, Hiroshima 739-8528, Japan; 2 Faculty of Human Life Sciences, Fuji Women's University, Ishikari 061-3204, Japan; 3 Iida Women's Junior College, Iida 395-0812, Japan; 4 Manda Fermentation Co., Ltd., Onomichi, Hiroshima 722-2192, Japan

**Keywords:** fermented plant product, succinate, mucins, deoxycholate, immunoglobulin A

## Abstract

‘*Manda Koso*’ is a commercial fermented plant product (FPP) made from 53 types of fruits and vegetables that have been fermented for >3 years and 3 months. We hypothesized that FPP intake improves the luminal environment of rats fed a high-fat diet. Thus, the present study examined the effects of consumption of 5% FPP diet for 3 weeks on colonic luminal parameters in rats fed a 30% beef tallow diet. Food intake and body weight gain were unaffected. Consumption of the FPP diet did not influence the proportions of *Bifidobacterium*, *Lactobacillus*, *Bacteroides*, *Prevotella* or *Clostridium* in cecal contents. However, the FPP diet caused a significant reduction (−88%) in the level of cecal succinate, a putative inflammatory signal (P<0.01), but did not affect the levels of *n*-butyrate, propionate, acetate and lactate. The fecal levels of deoxycholate and hyodeoxycholate, which are toxic bile acids, were also significantly reduced by the FPP diet (P<0.05). The FPP diet significantly increased fecal immunoglobulin A and mucins responsible for intestinal immune and barrier functions (P<0.05). The results suggest that the consumption of FPP is beneficial for the colonic luminal environment in rats fed a high-fat diet.

## Introduction

‘*Manda Koso*’ (Manda Fermentation Co., Ltd., Onomichi, Japan) is a fermented plant product (FPP) made of naturally fermented fruits, plant roots, cereals, marine algae and kokuto, a type of non-antifungal cane sugar. The raw ingredients are crushed and fermented by *Lactobacillus* and yeast generated spontaneously from raw materials at room temperature for 3 years and 3 months. The product is a well-known natural health food that is consumed in Japan. The FPP is a sweet, black-brown, paste-like substance comprising 36.9% water, 2.4% proteins and amino acids, 3.7% dietary fibers, 55.2% carbohydrates and 1.8% ash. The consumption of FPP is reported to reduce the fat content without affecting bone weight or strength in ovariectomized rats (1). The FPP also exhibits free radical scavenging activity (2). The consumption of FFP in fish decreases thiobarbituric-acid reactive substance levels in their tissues (3). Additionally, FPP intake has been recently suggested to improve feed efficiency and the intestinal histological status in broilers (4).

The consumption of certain dietary fibers, including inulin and oligosaccharides, increases the concentrations of intestinal immunoglobulin A (IgA) and mucins, which have roles in the maintenance of gut barrier function (5,6). Colon IgA levels are decreased in patients with ulcerative colitis (7). IgA production was recently suggested to be associated with a decreased incidence of colon cancer (8). The intestinal fermentation of dietary fibers and oligosaccharides is associated with the enhanced intestinal production of *n*-butyrate (9). Elevated intestinal production of *n*-butyrate by fermentation is associated with decreased risks of colon cancer and ulcerative colitis (10,11). Certain fibers and polyphenols are reported to reduce fecal secondary bile acids, such as deoxycholate and lithocholate; secondary bile acids, which are the highly cytotoxic intestinal microbial metabolites of primary bile acid that promote colon cancer development (12,13). A high-fat diet increases fecal secondary bile acids and the production of succinate, a putative pro-inflammatory signal, and decreases *n*-butyrate production (14–16). These alterations are believed to be associated with the increased risks of colon cancer and ulcerative colitis.

Due to the favorable effect of FPP intake on the intestinal histological status in broilers, as mentioned above (4), we hypothesized that FPP intake improves the colonic luminal environment of rats fed a high-fat diet. Therefore, the effects of FPP consumption was investigated on intestinal luminal variables, including microflora, fermentation, secondary bile acids, IgA, mucins and harmful enzymes in rats fed a high-fat diet.

## Materials and methods

### 

#### Materials

The FPP was obtained from Manda Fermentation Co., Ltd., and the chemical composition is shown in Table I.

#### Animals

Male Sprague-Dawley rats (3-week-old) were purchased from Hiroshima Laboratory Animal Centre (Hiroshima, Japan) and maintained according to the ‘Guide for the Care and Use of Laboratory Animals’ established by Hiroshima University; the study protocol was approved by the University Ethics Committee. The rats were individually housed in an air-conditioned room at 23–24°C with a 12-h light cycle (light, from 08:00 a.m. to 8:00 p.m.). Following acclimatization and feeding with a non-purified commercial rodent diet (moderate fat; Oriental Yeast Co., Ltd., Tokyo, Japan) for 7 days, 13 rats (mean body weight, 105 g) were divided into 2 groups with 6 or 7 rats in each. The compositions of the experimental diets are shown in Table II. The FPP was added to the diet at 7.9% (w/w) (5% on dry weight basis). The levels of dietary minerals and fibers in the FPP diet were adjusted by reducing the salt mixture and cellulose, respectively. The amounts of dietary fibers in the FPP were measured using the AOAC 2001.03 enzyme-gravimetric method in combination with high-performance liquid chromatography (17). Equal amounts of each experimental diet were incorporated daily into food cups at 7:00 p.m. (9, 10, 12, 14 and 15 g for days 1, 2–4, 5–7, 8–13 and 14–21, respectively) to ensure a standardized food intake. All the diet was consumed each day until the diet was served on the following day. The weight of the spilled diet was recorded daily and accounted for in the calculation of food intake. Feces were collected during the last 3 days. At the end of the 21-day feeding period, the rats were sacrificed by decapitation under diethyl ether anaesthesia. The liver, epididymal and perirenal adipose tissues and gastrocnemius muscle were excised rapidly and weighed. The cecum was excised, and its contents were immediately collected, weighed, and stored at −70°C until analysis.

#### Quantification analyses

Bacterial genomic DNA was extracted from cecal digesta using an Isofecal DNA extraction kit (Nippon Gene, Co., Ltd., Tokyo, Japan) according to the manufacturer's instructions. The cecal microflora was analyzed using a terminal restriction fragment length polymorphism method as described previously (18). Cecal organic acids were measured as described previously (19). Fecal acidic sterols were analyzed using an internal standard (nor-deoxycholic acid; Steraloids, Wilton, NY, USA) by gas chromatography as described previously. The total IgA concentration in feces was measured using an ELISA quantitation kit (Bethyl Laboratories Inc., Montgomery, TX, USA). Mucins were extracted according to the method of Bovee-Oudenhoven *et al* (20) and quantitated using a fluorometric assay (21). The activities of harmful fecal enzymes, such as tryptophanase, β-glucuronidase and β-glucosidase, were determined as described previously (22).

#### Statistical analysis

Data are expressed as mean ± standard error. Statistical analysis was performed by Student's t-test. P<0.05 was considered to indicate a statistically significant difference.

## Results

### 

#### Characteristics

Final body weight, total food intake, weights of tissues and fecal weight did not differ significantly between the groups (Table III). The data of cecal microflora and organic acids are shown in Table IV. The proportions of the cecal microflora examined were unaffected. The cecal level of succinate was markedly reduced in the FPP diet group (−88%, P<0.01), while the levels of other organic acids did not differ significantly between groups.

The fecal contents of deoxycholate and hyodeoxycholate acid were significantly lower in the FPP diet group (−50 and −56%, respectively, P<0.05, Table V), while those of cholate and lithocholate were not significantly different. Cecal levels of IgA and mucins were 1.9- and 3.2-fold significantly greater in the FPP diet group (+91 and +219%, respectively, P<0.05). Furthermore, the activity of fecal β-glucuronidase tended to be lower in the FPP diet group (−23%, P=0.073). The activities of the other enzymes did not differ significantly between the groups.

## Discussion

Notably, the cecal succinate level was markedly reduced by the FPP diet in the present study, whereas other organic acids were unaffected. To the best of our knowledge, this is the first evidence of the marked reduction of colonic succinate by dietary factor(s). A high-fat diet was recently found to increase colonic succinate production and decrease butyrate production together with low-grade inflammation (16). Succinate is considered an inflammatory and hypoxic signal; it stabilizes the transcription factor hypoxia-inducible factor-1α in specific tumors and activated macrophages, and stimulates dendritic cells via succinate receptor GPR91 (23). Succinate, produced abundantly by members of the family *Bacteroidaceae*, particularly *B. caccae*, is considered the ulcerogenic agent in dextran sulfate sodium colitis (24). Succinic acid has been reported to reduce the proliferation rate of the epithelial cells in the colon, as well as the crypt size (25). Succinic acid has been shown to inhibit the motility of the large intestine and to stimulate water secretion from the small intestine (25). Thus, the present findings raise the possibility that the suppression in cecal succinate by FPP intake is beneficial for the colon. However, further study is required to confirm this.

Another important finding was the marked reduction in fecal deoxycholate and hyodeoxycholate (cytotoxic bile acids) by FPP intake. Deoxycholate is considered cytotoxic to normal colonic crypt cells, resulting in increased compensatory proliferation of colonic epithelium cells, which is associated with an increased risk of colon cancer (26,27). Meanwhile, deoxycholate causes DNA damage and oxidative stress, and has pro-inflammatory activity by activating nuclear factor-κB (28). Our previous study found that a 0.5% dietary supplementation with certain polyphenols, particularly curcumin, significantly reduces these secondary bile acids in rats fed a high-fat diet (13). As the FPP diet used in the present study contains a small amount of polyphenols (Table I), it is required to determine whether the effects of the FPP diet are due to the polyphenols in the diet.

The FPP diet significantly increased fecal IgA and mucins, which are responsible for intestinal immune and barrier functions (5,6). Certain dietary fibers and oligosaccharides are reported to increase IgA and mucin levels (5,6). The FPP contains small amounts of dietary fibers (Table I). Therefore, it is required to determine whether these ingredients are responsible for the observed effects. As certain harmful enzymes are considered to be associated with colon cancer (15,29), the activities of such fecal enzymes were examined further. The FPP diet tended to reduce the activity of fecal β-glucuronidase, although not significantly. Thus, collectively, the FPP diet appeared to be favorable for the luminal environment of rats fed a high-fat diet.

In conclusion, the present study provides evidence that the FPP, *Manda Koso*, is a beneficial agent for the colonic luminal environment in rats fed a high-fat diet by reducing succinate and deoxycholate levels, and increasing IgA and mucins levels. However, further study is required to elucidate the underlying mechanisms by which the FPP exerts such effects and to identify the active compounds responsible.

## Figures and Tables

**Table I.** Chemical composition of FPP.

Composition per 100 g FPP	FPP
Nitrogen × 6.25, g	2.4
Carbohydrates, g	55.2
Glucose, g	18.6
Fructose, g	15.8
Maltose, g	0.23
Isomaltose, g	0.8
Dietary fibers, g	3.7
Ash, g	1.8
K, mg	530
Ca, mg	130
Mg, mg	54
Na, mg	49
P, mg	47
Fe, mg	3.2
Zn, mg	0.6
Water, g	36.9
Ile, mg	77
Leu, mg	142
Lys, mg	43
Met, mg	23
Phe, mg	83
Tyr, mg	44
Thr, mg	63
Try, mg	12
Val, mg	99
His, mg	23
Arg, mg	37
Ala, mg	86
Asp, mg	238
Glu, mg	336
Gly, mg	62
Pro, mg	115
Ser, mg	73
Vitamin B1, mg	0.01
Vitamin B2, mg	0.02
Vitamin B6, mg	0.16
Vitamin K1, µg	2
Folic acid, µg	11.5
Niacin, mg	0.73
Retinol, µg	7
α-Carotene, µg	5
β-Carotene, µg	85
Soy isoflavone, mg	1.3
Total polyphenols, g	0.48
Lactate, g	1.2
Acetate, g	0.3
Tartarate, g	0.01
Succinate, g	0.03
Gluconate, g	0.72

FPP, fermented plant product.

**Table II.** Composition of the experimental diets.

Composition g/100 g	Control	FPP
Beef tallow	30.0	30.0
Casein (net protein 17.4 g/100 g diet)	20.0	19.8
L-cystine	0.3	0.3
Vitamin mixture	1.0	1.0
Salt mixture	3.5	3.4
Cellulose	5.0	4.7
Sucrose	20.0	20.0
Corn starch	20.2	15.8
FPP (net content 5.0 g/100 g diet)	0.0	7.9

FPP, fermented plant product.

**Table III.** Body, tissue, cecal content and fecal weights.

Characteristics	Control	FPP
Final body weight, g	253±4	242±5
Total food intake, g	275±2	271±5
Liver weight, g	11.2±0.3	11.1±0.4
Epididymal adipose tissue weight, g	3.20±0.23	3.19±0.19
Perirenal adipose tissue weight, g	3.85±0.41	4.06±0.31
Gastrocnemius muscle weight, g	2.86±0.11	2.59±0.07
Weight of cecum contents, g	1.67±0.08	1.47±0.11
Fecal dry weight, g/3 days	3.44±0.15	3.35±0.32

Mean ± standard error (n=6–7). FPP, fermented plant product.

**Table IV.** Effect of consumption of fermented plant product (FPP) diet on cecal mucroflora and organic acids.

Characteristics	Control	FPP	Change, %
Cecal microflora, %			
*Bifidobacterium*	0.74±0.26	0.40±0.19	
*Lactobacillales*	16.36±6.18	10.76±3.42	
*Bacteroides*	21.60±4.51	22.76±4.14	
*Prevotella*	1.99±0.78	3.67±1.00	
*Clostridium cluster IV*	0.52±0.52	0.29±0.21	
*Clostridium subcluster XIVa*	12.50±1.69	13.90±1.28	
*Clostridium cluster XI*	12.39±1.15	13.60±1.80	
*Clostridium cluster XVIII*	4.70±0.91	3.77±1.24	
Others	29.19±2.62	30.85±2.69	
Cecal organic acids, µmol/total contents			
Succinate	9.3±2.9	1.1±0.3^a^	−88
Lactate	1.3±0.3	1.6±0.2	+23
Acetate	42.7±3.3	44.6±6.2	+4
Propionate	15.5±1.3	13.0±1.5	−16
*n*-Butyrate	15.9±2.0	10.7±2.1	−33
Total organic acids	84.7±6.8	70.2±9.7	−17

Mean ± standard error (n=6–7).

a P<0.05 by Student's t-test.

**Table V.** Effect of dietary FPP on fecal parameters in rats fed a high-fat diet.

Characteristics, amount/3 days	Control	FPP	Change, %
Lithocholate, µmol,	1.00±0.13	0.92±0.32	
Deoxycholate, µmol	1.87±0.23	0.93±0.26^a^	-50
Hyodeoxycholate, µmol	5.78±0.83	2.52±0.95^a^	-56
Cholate, µmol	0.54±0.20	0.18±0.09	
IgA, mg	0.89±0.05	1.70±0.31^a^	+91
Mucins, mg	1.18±0.12	3.76±0.38^a^	+219
Tryptophanase activity, U	0.27±0.06	0.90±0.56	
β-glucuronidase activity, U	3.28±0.27	2.51±0.33	
β-glucosidase activity, U	0.66±0.15	1.18±0.31	

Mean ± standard error (n=6–7).

a P<0.05 by Student's t-test (P<0.05).

## Abbreviations

FPP: fermented plant product
